# Imagery Rescripting Versus Cognitive Restructuring for Social Anxiety: Treatment Effects and Working Mechanisms

**DOI:** 10.32872/cpe.5303

**Published:** 2021-09-30

**Authors:** Miriam Strohm, Marena Siegesleitner, Anna E. Kunze, Thomas Ehring, Charlotte E. Wittekind

**Affiliations:** 1 Department of Psychology, LMU Munich, Munich, Germany; Philipps-University of Marburg, Marburg, Germany

**Keywords:** imagery rescripting, cognitive restructuring, social anxiety, mental imagery, working mechanisms, autobiographical memories

## Abstract

**Background:**

Negative mental images in social anxiety are often linked to memories of distressing social experiences. Imagery Rescripting (ImRs) has been found to be a promising intervention to target aversive memories, but mechanisms underlying ImRs are largely unknown. The present study aimed (a) to investigate the effects of ImRs compared to cognitive restructuring (CR) on social anxiety symptoms and (b) to extend previous research by examining whether ImRs works by fostering reappraisal of negative emotional self-beliefs.

**Method:**

Highly socially anxious individuals (N = 77) were randomly allocated to ImRs, CR, or no intervention control (NIC). A speech task was performed at baseline and at 1-week follow-up.

**Results:**

Only CR significantly reduced social anxiety symptoms from baseline to follow-up. Decreases in negative appraisals and emotional distress in response to the speech task did not differ between conditions. Regarding working mechanisms, ImRs led to stronger increases in positive emotions than CR and NIC. Both CR and ImRs yielded short-term reductions in emotionally anchored idiosyncratic self-beliefs, but CR was superior to ImRs at follow-up.

**Conclusions:**

The present study provides evidence for the efficacy of a single-session of CR for social anxiety symptoms. As one specific version of ImRs was applied, it is conceivable that other or optimized versions of ImRs might be more effective.

## Highlights

- CR was more effective than ImRs and no intervention to reduce social anxiety symptoms.
- CR more effectively reduced dysfunctional beliefs.
- ImRs led to strongest increase of positive emotions.

Cognitive models of social anxiety disorder (SAD) suggest that negative mental images of the self are a key maintaining factor of the disorder (Clark & Wells, 1995; Hofmann, 2007; Rapee & Heimberg, 1997). Image content is often linked to former aversive social experiences (Hackmann et al., 2000). Therefore, specifically targeting these aversive memories during treatment might improve therapeutic outcomes (Norton & Abbott, 2017; Wild & Clark, 2011).

Imagery Rescripting (ImRs) is an imagery-based intervention for aversive memories that has increasingly been incorporated in cognitive behavioral therapy (CBT) for SAD (e.g., McEvoy et al., 2020; McEvoy & Saulsman, 2014; Wild & Clark, 2011). During ImRs, patients are instructed to visualize an aversive memory and to change it in imagination according to their emotional needs. ImRs aims to update the meaning of memories thereby reducing associated negative (self-)images, beliefs, and emotions (Arntz, 2012). ImRs may be an efficacious treatment for different disorders including SAD (Morina et al., 2017). Several studies have found that one session of ImRs significantly improved social anxiety symptoms (Lee & Kwon, 2013; Wild et al., 2007, 2008), also when delivered as a stand-alone intervention and without prior cognitive restructuring (CR; Nilsson et al., 2012; Norton & Abbott, 2016; Reimer & Moscovitch, 2015). While ImRs yields promising treatment results, a better understanding of its underlying working mechanisms is needed to eventually optimize treatment efficacy.

It has been proposed that ImRs might work by changing the idiosyncratic meaning of aversive experiences (Arntz, 2012) and, more specifically, by leading to *emotionally anchored reappraisal* of core beliefs (Nilsson et al., 2012; Norton & Abbott, 2016; Wild et al., 2008). During ImRs, positive meanings are offered in the form of images. Based on evidence that mental imagery elicits stronger emotions than verbal thinking (Holmes & Mathews, 2010), it is conceivable that generating images with alternative meanings during ImRs is associated with stronger emotional activation than questioning maladaptive beliefs verbally (Holmes et al., 2009). Consequently, alternative meanings offered in the form of images might be more emotionally anchored, more believable, and more likely to lead to changes in behavior than meanings exclusively generated as verbal representations (Holmes & Mathews, 2010). This assumption is in line with the idea that one can distinguish between different levels of meaning representations (e.g., Barnard & Teasdale, 1991; but see Power & Dalgleish, 1999). According to the Model of Interacting Cognitive Subsystems (ICS; Barnard & Teasdale, 1991), intellectual beliefs (propositional level) can be distinguished from emotional beliefs (implicational level). Intellectual beliefs are described as knowing something “with the head”, whereas emotional beliefs correspond to an implicit sense of knowing “with the heart” or “having a gut feeling” (Barnard & Teasdale, 1991). Cognitive treatments can be expected to change beliefs primarily on a propositional level. ImRs as an experientially oriented intervention invokes different sensory modalities thereby addressing the implicational meaning level, which is suggested to be necessary to then change emotional beliefs (see Arntz, 2012; Wild et al., 2008). Although *emotionally anchored reappraisal* (i.e., changing emotional beliefs) has often been discussed as a mechanism underlying ImRs, empirical evidence is largely missing. One study with a sample of Bulimia Nervosa patients has investigated effects of ImRs on emotional vs. intellectual beliefs (Cooper et al., 2007). ImRs was found to be more effective than a control intervention in reducing emotional self-beliefs. A recent study investigated the effects of ImRs (vs. imaginal exposure [IE] and supportive counselling [SC]) on memory processes in patients with social anxiety disorder (Romano et al., 2020). There were no differences between conditions regarding memory appraisal, but a higher proportion of patients receiving ImRs updated their negative core belief compared to SC (no differences emerged compared to IE). Given the limited number of studies on working mechanisms of ImRs, the aim of the present study was to investigate whether ImRs works by reducing maladaptive emotional beliefs.

The present study aimed to (1) investigate the effects of stand-alone ImRs and CR on social anxiety symptoms, and (2) extend previous research by exploring mechanisms underlying ImRs. Our procedure was based on the study by Norton and Abbott (2016). Highly socially anxious individuals were randomly allocated to either one session of ImRs, one session of CR, or a no-intervention control condition (NIC). Outcomes were assessed at baseline and at 1-week follow-up. A speech task was included to examine intervention effects to a social stressor. In line with previous findings, we hypothesized that ImRs and CR would yield greater decreases in social anxiety symptoms than NIC. We expected ImRs and CR to reduce negative appraisals and emotional responses (subjective arousal and distress) to the speech task more strongly than NIC. Regarding mechanisms, we hypothesized that ImRs would lead to stronger emotional activation than CR. While we expected both ImRs and CR to decrease the maladaptive intellectual self-beliefs, we assumed that ImRs would yield stronger reductions of maladaptive emotional self-beliefs. We additionally explored the relationship between the hypothesized mechanisms and symptomatic change.

## Method

### Participants

Highly socially anxious individuals were recruited via advertisements on university campus and social media. To be included, participants had to score ≥ 30 (clinical cut-off) on the German version of the Social Interaction Anxiety Scale (SIAS; Stangier et al., 1999). Results of a sample-size calculation (two-tailed, α = .05, power = .80, run with G*Power 3.1; Faul et al., 2007) with medium to large effect sizes (*d* = .70; Morina et al., 2017) showed that a sample size of 76 was required to detect significant differences between active treatments (ImRs + CR) versus NIC.

During the first session, eligible participants were administered the Mini International Neuropsychiatric Interview (M.I.N.I. 5.0.0; Sheehan et al., 1998; German version: Ackenheil et al., 1999) to screen for exclusion criteria: (1) current diagnosis of Major Depressive Disorder, (2) current and/or lifetime diagnosis of Posttraumatic Stress Disorder/Psychotic Disorder/Bipolar Disorder, (3) Substance Dependence during the past 12 months, (4) acute suicidal tendencies. Further exclusion criteria were: (5) age < 18 or > 35 years, (6) current psychological treatment, (7) pregnancy, (8) severe physical illness. The restricted age range was applied to obtain a more homogenous sample regarding age. Participants had to meet the following inclusion criteria: (1) negative mental self-image(s) in feared social situations, (2) aversive social experience related to the image, and (3) maladaptive self-belief (see Section "Imagery Interview").

A total of 96 participants attended Session 1 of whom 16 had to be excluded (*n* = 10 current/lifetime diagnosis of mental disorders specified above; *n* = 4 no negative mental self-image; *n* = 2 no maladaptive self-belief). Three participants did not attend the follow-up session, leaving a final sample of 77 participants (81% female; age: *M* = 22.46, *SD* = 3.88). All participants gave written informed consent and were reimbursed by receiving partial course credit or 20€. The study was approved by the Research Ethics Committee of the Faculty of Psychology and Educational Sciences at LMU Munich.

### Clinical Interviews

The M.I.N.I. (Sheehan et al., 1998; German version: Ackenheil et al., 1999) was administered to assess current diagnoses according to DSM-IV (American Psychiatric Association [APA], 2000). Additionally, the SAD module of the Structured Clinical Interview for DSM-IV (SCID-I; First et al., 2002; German version: Wittchen et al., 1997) was administered.

### Imagery Interview

The Imagery Interview was based on the Waterloo Images and Memories Interview (WIMI; Moscovitch et al., 2011) and on the interview used by Norton and Abbott (2016). The semi-structured interview assessed negative self-imagery, aversive memories, and maladaptive self-beliefs. Participants were asked to define their most anxiety-provoking social situation and to imagine themselves being in such a situation. They were instructed to become aware of whether there was a mental image that comes to their mind in this kind of situation and to describe the mental image in detail. Participants were then asked when they first felt the way they did in the image and to visualize and describe the respective event. This was used to determine whether there was an early aversive memory related to the mental image. In order to specify the idiosyncratic self-belief derived from the negative mental image and the aversive memory, participants were asked: “What do the image and the memory tell about you as a person?”. Participants were instructed to summarize the meaning in form of a short statement.

### Speech Task

In order to measure reactions to a social stressor, participants were asked to give a 3 min video-recorded impromptu speech (Norton & Abbott, 2016) on a given political topic in both sessions (the order of two topics was counterbalanced).

### Symptom Measures

The 20-item SIAS (Mattick & Clarke, 1998; German version: Stangier et al., 1999) was used to assess social interaction anxiety during the past seven days on a 5-point scale (0 = *not at all* to 4 = *extremely*). The 12-item Brief Fear of Negative Evaluation Scale-Revised (BFNE-R; Carleton et al., 2006; German version: Reichenberger et al., 2016) was administered to measure fear of negative evaluation by others on a 5-point scale (1 = *not at all characteristic of me* to 5 = *extremely characteristic of me*). In order to test for baseline group differences in depressive symptoms, the Patient Health Questionnaire-9 Item (PHQ-9; Krönke et al., 2001; German version: Löwe et al., 2002) was administered.

### Speech Task Measures

In order to verify the relevance of the speech task as a stressor we asked participants to indicate how anxious they had felt or would have felt when giving a speech/presentation during the last week (0 = *not at all anxious* to 3 = *extremely anxious*). The Probability and Consequences Questionnaire (PCQ; Rapee & Abbott, 2007) asks participants to rate their appraisal of the likelihood (7 items) and cost (7 items) of negative evaluation of their speech on a 5-point scale (0 = *not at all likely/bad* to 4 = *extremely likely/bad*). Subjectively experienced levels of distress were assessed using Subjective Units of Distress (SUD, 0 = *not at all distressed* to 100 = *extremely distressed*). Self-assessment manikins (SAM; Bradley & Lang, 1994) were used to assess self-reported physiological arousal (1 = *very calm* to 9 = *very aroused*).

### Measures of Underlying Mechanisms

#### Emotional Activation

The Positive and Negative Affect Schedule-Extended (PANAS-X; Watson & Clark, 1994; German version: Grühn et al., 2010) was administered to assess changes in positive and negative emotions from pre- to post-intervention. Participants were instructed to indicate how they felt at this very moment. We included the general dimensions “positive affect” (PA) and “negative affect” (NA) as well as the subscales “fear”, “hostility”, “guilt”, “sadness”, “joviality”, “self-assurance”, and “attentiveness”. Scales range from 1 (*very slightly or not at all*) to 5 (*extremely*).

#### Intellectual and Emotional Beliefs

The maladaptive self-belief was identified during the Imagery Interview. Participants were asked to rate intellectually and emotionally how much they felt that this belief was true (see Cooper et al., 2007). For the intellectual rating, participants were asked to indicate how much they would rationally agree to their belief (0 *= I do not agree at all* to 100 = *I completely agree).* For the emotional rating, participants were asked how much they *felt* the belief was true, regardless of what they were thinking rationally (0 *= feels not true at all* to 100 = *feels completely true)*.

### Interventions

#### Imagery Rescripting

The ImRs procedure was based on protocols by Arntz and Weertman (1999) and Wild and Clark (2011). Stage 1 of ImRs started with participants closing their eyes and vividly imagining the aversive memory from the perspective of their younger-self. Participants were instructed to describe the situation in the first person, present tense, and to include all sensory modalities. Stage 2 of ImRs was initiated by instructing participants to imagine the scene from the perspective of their current adult-self who is witnessing the events as a bystander. Participants were asked to describe what they see is happening to their younger-self and were then encouraged to intervene in any way they wished. When the adult-self felt fully satisfied, Stage 3 was initiated by asking participants to relive the memory again from the perspective of their younger-self, experiencing the interventions of their adult-self. Additionally, the younger-self was encouraged to express further unmet needs. The ImRs procedure was concluded by asking participants to dwell on the final positive image. As we wanted to elucidate the underlying mechanisms of ImRs (vs. CR) on symptom change, we used “pure” interventions and tested ImRs in isolation. Consequently, ImRs was not preceded by cognitive restructuring and we did not explicitly refer to the maladaptive self-belief during ImRs. The mean duration of ImRs was 22.35 min (*SD* = 6.20).

#### Cognitive Restructuring

The CR procedure was based on the protocol by Wild and Clark (2011). Participants were first asked to outline evidence for their maladaptive self-belief and were then encouraged to challenge the self-belief by collecting evidence against it. To support this process we asked participants to consider alternative explanations for their experiences (including the early aversive memory), and to think of experiences contradicting the self-belief. All evidence for and against the negative self-belief was written down on a worksheet. Finally, participants were instructed to rephrase the original self-belief into a more helpful statement. The mean duration of CR was 23.74 min (*SD* = 4.40).

#### No-Intervention Control Condition

Participants in NIC were provided neutral magazines and were instructed to wait for 30 min in the laboratory. They were asked not to use any electronic device.

### Procedure

The study comprised two sessions, which were one week apart. Two experimenters carried out different parts of the procedure so that the speech task and intervention were not administered by the same experimenter. During Session 1, Experimenter 1 administered the clinical interviews and baseline measurements (t0: sociodemographic data, SIAS, BFNE-R, public speaking anxiety, SUIS, ERQ), followed by pre-speech measures (SUD, SAM, PCQ) and the speech task. Experimenter 2 then conducted the Imagery Interview and administered pre-treatment questionnaires (t1: intellectual and emotional belief, PANAS-X). Then, participants were randomly allocated to ImRs (*n* = 25), CR (*n* = 27), or NIC (*n* = 25). The allocation sequence was computer-generated and Experimenter 2 was blinded until the beginning of the interventions, Experimenter 1 was blinded during the entire study. Immediately after the interventions or the waiting period, participants completed post-treatment measures (t2: intellectual and emotional belief, PANAS-X). During Session 2, which took place one week later, Experimenter 1 administered the follow-up questionnaire (t3: SIAS, BFNE-R, intellectual and emotional belief) and the second speech task, again including speech task measures administered prior to the speech task (SUD, SAM, PCQ). Finally, participants were fully debriefed.

### Statistical Analyses

A series of 2(Time) x 3(Condition) repeated measures ANOVAs were carried out for social anxiety symptoms (t0; t3), for speech task measures (pre-speech1; pre-speech2), and for positive and negative emotions (t1; t2). To follow up significant interactions, planned contrasts on change scores were conducted (ImRs+CR vs. NIC; ImRs vs. CR). Effects on intellectual and emotional self-beliefs were tested with 3(Time) x 3(Condition) repeated measures ANOVAs. Significant interactions were followed up using planned contrasts (ImRs+CR vs. NIC; ImRs vs. CR). For ImRs, Pearson correlations were computed between mechanisms and symptomatic change. A significance level of α = .05 (two-tailed) was used for all analyses. Partial eta squared ($\eta_{p}^{2}$) or Cohen’s *d* were used as effect sizes.

## Results

### Participant Characteristics and Baseline Comparisons

No significant baseline differences between conditions emerged (see Table 1). Mean age at time of the aversive event was 12.86 years (*SD* = 4.55; range 3-27), with significant differences between groups^1^1We tested whether age of the aversive memory (i.e., time that had passed since the event) had an influence on our main symptomatic outcomes. However, results remained unchanged when including age of the memory as a covariate. Note that age of the aversive memory was not significantly different in the two active treatment conditions (ImRs and CR). (ImRs: *M* = 13.88, *SD* = 4.90; CR: *M* = 13.76, *SD* = 4.60; NIC: *M* = 10.88, *SD* = 3.55), *F*(2, 74) = 3.78, *p* = .027.

**Table 1:** Demographic Variables and Pre-Treatment Characteristics

Demographics and pre-treatment characteristics	Overall sample (*N* = 77)	ImRs (*n* = 25)	CR (*n* = 27)	NIC (*n =* 25)	Statistics
Demographics					
Gender (female/male), *n*	62/15	21/4	20/7	21/4	χ^2^(2) = 1.10, *p* = .577
Age in years, *M* (*SD*)	22.36 (3.88)	22.64 (3.82)	22.59 (3.92)	21.84 (4.01)	*F*(2,74) = 0.33, *p* = .718
Social anxiety symptoms, *M* (*SD*)					
SIAS	40.29 (12.55)	40.84 (13.21)	37.93 (12.06)	42.28 (12.49)	*F*(2,74) = 0.81, *p* = .447
BFNE-R	40.48 (10.39)	40.20 (11.00)	39.44 (10.36)	41.88 (10.07)	*F*(2,74) = 0.36, *p* = .696
**SAD Criteria met, *n* (%)**	21 (27)	8 (32)	8 (30)	5 (20)	χ^2^(2) = 1.02, *p* = .599
Comorbidity (yes/no), *n*	7/70	3/22	3/24	1/24	
Generalized Anxiety Disorder, *n*	2	0	1	1	
Dysthymia	3	1	2	0	
Anorexia Nervosa	1	1	0	0	
Bulimia Nervosa	1	1	0	0	
**Public Speaking Anxiety, *M* (*SD*)**	1.94 (0.85)	1.92 (0.95)	1.93 (0.96)	1.96 (0.61)	*F*(2,74) = 0.02, *p* = .984

*Note.* ImRs = Imagery Rescripting; CR = Cognitive Restructuring; NIC = No-Intervention Control; SIAS = Social Interaction Anxiety Scale; BFNE-R = Brief Fear of Negative Evaluation Scale-Revised; SAD = Social Anxiety Disorder.

### Social Anxiety Symptoms

#### Social Interaction Anxiety

**Figure 1:**
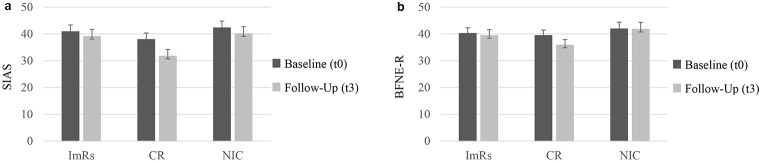
Effects of ImRs vs. CR vs. NIC on (a) Social Interaction Anxiety (SIAS), and (b) Fear of Negative Evaluation (BFNE-R) *Note.* Error Bars Represent SEM.

For SIAS scores (see Figure 1), there was no main effect of Condition, *F*(2, 74) = 1.97, *p* = .147, $\eta_{p}^{2}$ = .05, but a significant effect of Time, *F*(1, 74) = 17.94, *p* < .001, $\eta_{p}^{2}$ = .20, and a significant interaction, *F*(2, 74) = 3.22, *p* = .046, $\eta_{p}^{2}$ = .08. Planned contrasts revealed no difference between the active treatment groups compared to NIC in reducing social interaction anxiety, *t*(74) = 1.05, *p* = .298, *d* = 0.26. However, CR led to stronger decreases than ImRs, *t*(74) = 2.29, *p* = .025, *d* = 0.64.

#### Fear of Negative Evaluation

Results for BFNE-R revealed a significant main effect of Time, *F*(1, 74) = 5.70, *p* = .020, $\eta_{p}^{2}$ = .07, but neither a significant effect of Condition, *F*(2, 74) = 1.09, *p* = .342, $\eta_{p}^{2}$ = .03, nor a significant interaction, *F*(2, 74) = 2.90, *p* = .061, $\eta_{p}^{2}$ = .07., see Figure 1.

### Speech Task Measures

For both subscales of the PCQ^2^2In some participants, speech-related questionnaires were erroneously not administered (PCQ: *n* = 3; SUD: *n* = 4; SAM: *n* = 2) and these participants were excluded from the respective analyses., there were significant main effects of Time, *F*s(1, 71) > 9.74, *p*s < .003, $\eta_{p}^{2}$s ≥ .12, but no significant interactions, *F*s(2, 71) < 2.28, *p*s > .110, $\eta_{p}^{2}$s ≤ .06. The main effect of Condition was significant for probability, *F*(2, 71) = 3.13, *p* = .050, $\eta_{p}^{2}$ = .08, but not for cost of negative evaluation, *F*(2, 71) = 1.13, *p* = .330, $\eta_{p}^{2}$ = .03. ImRs and CR did not yield significantly greater reductions of appraisals of negative evaluation than NIC (see Table 2).

**Table 2:** Means and Standard Deviations for Speech Task Measures Before (Speech 1) and After (Speech 2) Intervention: Means (SD)

Group	Speech 1	Speech 2
	*M* (*SD*)	*M* (*SD*)
Negative Evaluation: Probability^a^		
ImRs	15.46 (4.25)	14.79 (4.66)
CR	13.04 (5.62)	10.27 (5.45)
NIC	14.25 (5.93)	13.33 (5.93)
Negative Evaluation: Cost^a^		
ImRs	13.50 (5.87)	12.25 (6.10)
CR	12.46 (6.71)	9.27 (4.64)
NIC	13.79 (5.98)	12.29 (6.52)
Distress (SUD)^b^		
ImRs	66.50 (29.77)	57.42 (27.33)
CR	75.12 (22.01)	55.35 (28.42)
NIC	72.17 (23.10)	65.65 (24.33)
Arousal (SAM)^c^		
ImRs	6.67 (1.61)	5.79 (1.35)
CR	6.62 (1.50)	5.65 (1.67)
NIC	6.28 (1.67)	6.00 (1.61)

*Note.* ImRs = Imagery Rescripting; CR = Cognitive Restructuring; NIC = No-Intervention Control; SUD = Subjective Units of Distress; SAM = Self-Assessment Manikins.

^a^*n* = 74. ^b^*n* = 73. ^c^*n* = 75.

For distress (SUD), a significant effect of Time emerged, *F*(1, 70) = 17.41, *p* < .001, $\eta_{p}^{2}$ = .20, but neither the main effect of Condition nor the interaction were significant, *F*s(2, 70) < 2.12, *p*s > .128, $\eta_{p}^{2}$s < .06 (see Table 2).

Results for arousal (SAM) revealed a significant effect of Time, *F*(1, 72) = 11.35, *p* = .001, $\eta_{p}^{2}$ = .14, but neither a significant main effect of Condition nor a significant interaction, *Fs*(2, 72) < 1.05, *p* > .354, $\eta_{p}^{2}$ < .03 (see Table 2).

### Mechanisms

#### Activation of Positive and Negative Emotions

For PANAS-PA and NA (see Table 3) there were significant effects of Time, *F*s(1, 74) > 35.10, *p*s < .001, $\eta_{p}^{2}$s ≥ .32, but no significant effects of Condition, *F*s(2, 74) < 2.17, *p*s > .121, $\eta_{p}^{2}$s ≤ .06. No significant interaction was found for PANAS-NA, *F*(2, 74) = 0.57, *p* = .570, $\eta_{p}^{2}$ = .02. A significant interaction emerged for PANAS-PA, *F*(2, 74) = 9.29, *p* < .001, $\eta_{p}^{2}$ = .20. Planned contrasts revealed that active treatments increased positive emotions more strongly than NIC (*M_diff_* = -0.52, *SD* = 4.48), *t*(60.89) = 3.97, *p* < .001, *d =* 0.97, with ImRs (*M_diff_* = -7.36, *SD* = 6.81) leading to stronger increases than CR (*M_diff_* = -3.52, *SD* = 5.35), *t*(45.54) = 2.25, *p* = .029, *d =* 0.62. Results for the remaining subscales of PANAS-X are provided in the Supplementary Materials (Table S1).

**Table 3:** Symptom Measures and Mechanism Variables Before the Interventions (t0/t1), After the interventions (t2) and at Follow-up (t3): Means (SD)

Group	t0/t1	t2	t3
	*M* (*SD*)	*M* (*SD*)	*M* (*SD*)
PANAS-PA			
ImRs	23.12 (5.20)	30.48 (8.21)	
CR	22.89 (6.79)	26.41 (7.12)	
NIC	22.92 (6.13)	23.44 (6.89)	
PANAS-NA			
ImRs	19.04 (6.77)	13.92 (3.64)	
CR	18.19 (6.29)	14.41 (5.37)	
NIC	18.60 (5.58)	13.48 (3.12)	
Intellectual belief			
ImRs	51.60 (27.53)	39.40 (26.91)	48.80 (26.55)
CR	64.74 (29.83)	40.37 (25.79)	42.52 (29.49)
NIC	57.8 (33.32)	55.48 (32.32)	57.24 (29.63)
Emotional belief			
ImRs	90.40 (10.88)	62.52 (19.71)	73.80 (18.10)
CR	84.07 (16.82)	56.11 (29.00)	52.78 (27.92)
NIC	83.08 (20.92)	81.36 (21.90)	79.60 (20.74)

*Note.* ImRs = Imagery Rescripting; CR = Cognitive Restructuring; NIC = No-Intervention Control; SIAS = Social Interaction Anxiety Scale; BFNE-R = Brief Fear of Negative Evaluation Scale-Revised; PANAS = Positive and Negative Affect Schedule; PA = positive affect; NA = negative affect.

#### Intellectual and Emotional Beliefs

To check whether participants were able to distinguish between the intellectual and the emotional belief, a correlation between the two measures was computed. The moderate correlation of *r*_s_ = .387, *p* = .001, suggests that the two measures have some overlap but are not identical. For intellectual beliefs, there was no significant effect of Condition, *F*(2, 74) = 1.00, *p* = .373, $\eta_{p}^{2}$ = .03, but a significant effect of Time and a significant interaction, *F*s(1.81, 134.19 / 3.63, 134.19) > 6.12, *p*s < .001, $\eta_{p}^{2}$s ≥ .14 (see Table 3). Planned contrasts revealed that active treatments led to stronger reductions in intellectual beliefs from pre- to post-intervention than NIC, *t*(55.43) = 4.58, *p* < .001, *d =* 1.12, and from pre to follow-up, *t*(74) = 2.13, *p* = .036, *d =* 0.52. CR led to stronger reductions than ImRs from pre- to post-intervention, *t*(35.93) = 2.03, *p* = .050, *d =* 0.49, and from pre to follow-up, *t*(74) = 3.04, *p =* .003, *d =* 0.84.

For emotional beliefs, there were significant effects of Time and Condition, *F*s(2, 148/2, 74) > 5.37, *p*s ≤ .006, $\eta_{p}^{2}$s ≥ .13, and a significant interaction, *F*(4, 148) = 13.94, *p* < .001, $\eta_{p}^{2}$ = .27. Planned contrasts revealed that the active treatments reduced emotional beliefs more strongly than NIC from pre- to post-intervention, *t*(60.66) = 8.51, *p* < .001, *d* = 2.07, and from pre to follow-up, *t*(69.14) = 5.62, *p* < .001, *d* = 1.37. CR and ImRs decreased emotional beliefs from pre- to post-intervention equally effective, *t*(49.78) = -0.16, *p* = .878, *d =* 0.04, but CR led to stronger reductions than ImRs from pre to follow-up, *t*(48.13) = 2.67, *p* = .010, *d =* 0.74.

#### Correlations Between Mechanisms and Symptomatic Change

Within the ImRs group, symptomatic change was not significantly correlated with changes in emotions (PA x SIAS: *r* = -.08; PA x BFNE-R: *r* = .26; NA x SIAS: *r* = -.35; NA x BFNE-R: *r* = .11; *p*s ≥ .085) nor with pre-post changes in emotional beliefs and symptomatic change (SIAS: *r* = -.39; BFNE-R: *r* = -.15; all *p*s ≥ .055). The same non-significant pattern emerged in the CR group (PA x SIAS: *r* = -.25; PA x BFNE: *r* = .07; NA x SIAS: *r* = -.13; NA x BFNE: *r* = -.12; rational belief x SIAS: *r* = .14; rational belief x BFNE: *r* = .09, *p*s ≥ .217).

## Discussion

The present study examined the effects of single-session ImRs vs. CR for socially anxious individuals compared to NIC.

### Effects on Social Anxiety Symptoms

Contrary to hypothesis, we found that one session of cognitive restructuring (CR) is more effective than one session of imagery rescripting (ImRs) and no intervention control (NIC) in reducing social interaction anxiety. No significant differences between groups emerged for fear of negative evaluation. When confronted with the speech task, participants in all conditions demonstrated equal reductions in distress, arousal, and negative appraisals suggesting that if CR and ImRs are administered as very brief interventions no beneficial effects emerge over and above mere exposure to the speech. The speech task represents a strength of the study, but our findings suggest that the speech task may be susceptible to exposure effects, thereby reducing its ability to capture between-group differences in anxiety across time. Taken together, we could not replicate previous findings regarding the effects of the interventions on responses to a social stressor (Norton & Abbott, 2016). Our findings support previous evidence that one session of CR exerts positive effects on social anxiety symptoms (e.g., Norton & Abbott, 2016; Shikatani et al., 2014). Contrary to expectations, we were not able to replicate earlier findings on the benefits of stand-alone ImRs (Nilsson et al., 2012; Norton & Abbott, 2016; Reimer & Moscovitch, 2015) on social anxiety symptoms. This result is surprising given the similarities between studies (i.e., one session of ImRs, no cognitive preparation); however, a sub-clinical sample was included in our study whereas participants were diagnosed with SAD in previous research (Nilsson et al., 2012; Norton & Abbott, 2016; Reimer & Moscovitch, 2015). Although the severity of self-reported interaction anxiety in our study was comparable to previous studies (ø40 [this study]; ø37 [Nilsson et al, 2012]; ø44 [Norton & Abbott, 2016]), the low rate of diagnoses in the present sample could indicate that the impairment caused by the social anxiety symptoms was not sufficient to fulfill diagnostic criteria and that participants are able to cope with their negative mental images.

As our ImRs procedure closely followed the procedure of Norton and Abbott (2016), it seems rather unlikely that procedural differences explain the inconsistent findings. Alternatively, ImRs as used in this study might need to be optimized. First, ImRs might not have been optimally delivered (e.g., insufficient reactivation of emotions or the hotspot; short duration of ImRs [ø 22min in the present study]). Second, we do not know to what extent participants were able to put themselves in their younger self´s perspective. Third, in order to ensure internal validity we used a highly standardized ImRs protocol whereas other studies administered ImRs in a more individualized way and with a more active therapist/ experimenter (e.g., Norton & Abbott, 2016). Fourth, participants were instructed to introduce changes themselves in the present study. Finally, as dysfunctional self-beliefs were not explicitly addressed during ImRs it cannot be ruled out that the rescripting did not show a good enough match with the dysfunctional self-beliefs in the sense of providing corrective information and experiences to modify this belief. This may provide another explanation why ImRs was not associated with long-term effects in our study. Therefore, as the ImRs protocol used in the present study represents only one specific implementation of ImRs, it is conceivable that other versions of ImRs might have yielded more stable effects. For example, in accordance with the protocol by Wild and Clark (2011), a combination of ImRs with CR (Lee & Kwon, 2013; Wild et al., 2008) might yield more stable treatment effects. Different ImRs techniques have been applied in both research and clinical practice; however, it remains an open question how ImRs is best realized (e.g., with or without cognitive preparation; active vs. passive role of patient/therapist), therefore, future research is clearly needed to identify the most effective implementation of ImRs.

### Mechanisms Underlying Imagery Rescripting

In line with our hypothesis and with previous evidence (Holmes & Mathews, 2010), a single session of ImRs led to stronger increases of positive emotions than CR and NIC. In contrast, negative emotions significantly decreased across time with no differences between conditions. ImRs and CR more strongly reduced maladaptive intellectual and emotional beliefs from pre- to post-intervention compared to NIC, but only for CR reductions remained stable across time. In ImRs, neither changes in positive emotions nor in emotional beliefs correlated with symptomatic outcomes.

Although our results indicate that brief ImRs led to beneficial (short-term) effects, it remains to be tested whether the aforementioned mechanisms play a role in producing symptomatic change, as ImRs did not yield improvements on symptom measures in the present study. Moreover, our results challenge the notion that emotionally anchored reappraisal is a mechanism specific to ImRs. In fact, brief CR seems to be more effective in targeting maladaptive emotional beliefs in the longer-term, counter to the theoretical idea that cognitive treatment strategies primarily change intellectual meaning levels (i.e., propositional level). However, after a single session of CR mean levels of emotional beliefs were still high at follow-up and more systematic research is needed to test whether emotional beliefs can be further reduced with multiple treatment sessions.

### Limitations

ImRs and CR were delivered as very brief interventions within a non-therapeutic setting. Thus, the interventions deviate from treatment as used in clinical practice limiting its generalizability. However, laboratory-based studies in healthy or subclinical samples are a valuable means to investigate mechanisms involved in psychological treatments under highly controlled and standardized conditions (e.g., Van Den Hout et al., 2017). Although we inquired about the meaning of the mental image, we did not assess how distressing and how relevant the image was regarding participants´ social anxiety symptoms. The distress/impairment caused by the image should be inquired in future studies as it is conceivable that only the modification of distressing images might be associated with long-term effects on social anxiety symptoms. Moreover, it remains unclear whether participants adhered to the ImRs instructions and how distressed they were during ImRs as distress during ImRs was not assessed. Therefore, we cannot verify the correct implementation of ImRs and that emotional activation was sufficient. Emotional beliefs were rated on a one-item VAS, which might reduce reliability.

### Conclusion

The present study compared the effects of ImRs vs. CR as stand-alone single-session interventions in socially anxious individuals and aimed to examine mechanisms underlying symptomatic change. Results indicate that a single session of CR effectively reduces social anxiety symptoms. The present study raises the question how ImRs for socially anxious individuals should optimally be implemented in order to yield symptomatic change. We propose that more individualized ImRs protocols, higher treatment intensity, cognitive preparation, and/or directly targeting dysfunctional self-beliefs might be necessary to yield therapeutic effects.

## Supplementary Materials

The supplementary material contains a table containing the means and standard deviations of the positive and negative emotions as well as the results of the statistical analyses (for access see Index of Supplementary Materials below).

10.23668/psycharchives.5098 Supplement 1 Supplementary materials to "Imagery rescripting versus cognitive restructuring for social anxiety: Treatment effects and working mechanisms" [Additional results]

StrohmM.
SiegesleitnerM.
KunzeA. E.
EhringT.
WittekindC. E.
 (2021). Supplementary materials to "Imagery rescripting versus cognitive restructuring for social anxiety: Treatment effects and working mechanisms"
[Additional results]. PsychOpen. 10.23668/psycharchives.5098
PMC966723436398099

## Author Note

This paper was part of Miriam Strohm´s Ph.D. project (Strohm, 2019).
