# Supplementary material for: Imagery Rescripting Versus Cognitive Restructuring for Social Anxiety: Treatment Effects and Working Mechanisms
Source: Clin Psychol Eur. 2021 Sep 30;3(3):e5303. doi: 10.32872/cpe.5303 (PMC9667234; doi:10.32872/cpe.5303)
Supplement: Supplement 1 [file cpe-03-5303-s01.pdf]

SUPPLEMENTARY MATERIAL

Imagery Rescripting versus Cognitive Restructuring for Social Anxiety: Treatment Effects and Working Mechanisms

Miriam Strohm, Marena Siegesleitner, Anna E. Kunze, Thomas Ehring, &
Charlotte E. Wittekind*[1]

LMU Munich, Department of Psychology, Leopoldstraße 13, 80802 Munich, Germany

Manuscript published in Clinical Psychology in Europe

https://doi.org/10.32872/cpe.5303

*Correspondence should be addressed to Charlotte E. Wittekind, Department of Psychology, LMU Munich, Leopoldstraße 13, 80802 Munich, Germany, charlotte.wittekind@psy.lmu.de, +49 (0)89 2180 5196.

Table S1.
Positive and Negative Emotions: Means, Standard Deviations, Main Effects, and Interaction Effects.

	Group	Pre (t1)	Post (t2)	Main effect		Interaction effect
		M (*SD*)	*M* (*SD*)	Condition $F(1, 74)$	Time $F(1, 74)$	Condition x Time $F(2, 74)$
PANAS-X: Basic negative emotion						
Fear	ImRs	13.32 (4.88)	9.32 (3.06)	0.25	74.36***	0.18
	CR	12.56 (4.50)	9.07 (3.28)			
	NIC	13.48 (4.26)	9.40 (3.35)			
Hostility	ImRs	7.64 (2.46)	7.08 (2.36)	1.17	11.28***	0.59
	CR	8.04 (3.78)	6.75 (2.30)			
	NIC	7.08 (1.73)	6.12 (0.33)			
Guilt	ImRs	12.36 (4.26)	7.88 (1.76)	0.17	37.00***	2.90 (p=.061)
	CR	10.78 (4.14)	8.93 (3.85)			
	NIC	10.76 (4.88)	8.44 (2.87)			
Sadness	ImRs	9.72 (4.34)	7.12 (2.86)	0.02	37.02***	0.40
	CR	9.30 (4.14)	7.48 (3.86)			
	NIC	9.44 (4.22)	7.08 (2.10)			
PANAS-X: Basic positive emotion						
Joviality	ImRs	14.68 (5.07)	22.72 (7.92)	2.06	71.39***	3.41*
	CR	14.56 (6.24)	19.81 (7.68)			
	NIC	13.61 (5.04)	17.36 (5.23)			

Table S1 (continued)

	Group	Pre (t1)	Post (t2)	Main effect		Interaction effect
		M (*SD*)	*M* (*SD*)	Condition *F*(1, 74)	Time *F*(1, 74)	Condition x Time *F*(2, 74)
Self-assurance	ImRs	9.84 (3.27)	15.52 (5.11)	2.35	61.23***	13.32***
	CR	10.85 (4.99)	13.89 (4.89)			
	NIC	10.12 (3.46)	10.72 (4.03)			
Attentiveness	ImRs	11.80 (2.18)	13.16 (2.98)	0.54	0.00	8.24***
	CR	11.74 (2.68)	11.89 (2.79)			
	NIC	12.64 (3.17)	11.08 (3.25)			

Note: PANAS-X = The Positive and Negative Affect Schedule -Extended; ImRs = Imagery Rescripting (n=25); CR = Cognitive restructuring (n=27); NIC = No-intervention control condition (n=25). *p<0.05; *** p≤0.001.
